# Nutritional Status among Portuguese and Turkish Older Adults Living in the Community: Relationships with Sociodemographic, Health and Anthropometric Characteristics

**DOI:** 10.3390/nu15061333

**Published:** 2023-03-09

**Authors:** Meryem Elif Öztürk, Rui Poínhos, Cláudia Afonso, Nurcan Yabancı Ayhan, Maria Daniel Vaz de Almeida, Bruno M. P. M. Oliveira

**Affiliations:** 1 Department of Nutrition and Dietetics, Faculty of Health Sciences, Karamanoğlu Mehmetbey University, İbrahim Öktem Cd., 70100 Karaman, Turkey; 2 Faculty of Nutrition and Food Sciences, University of Porto, Rua do Campo Alegre, 823, 4150-180 Porto, Portugal; 3 Department of Nutrition and Dietetics, Faculty of Health Sciences, Ankara University, Fatih Caddesi 197/7, 06290 Ankara, Turkey; 4 GreenUPorto—Sustainable Agrifood Production Research Center, Rua da Agrária 747, 4485-646 Vairão, Portugal; 5 Artificial Intelligence and Decision Support, Institute for Systems and Computer Engineering—Technology and Science, Rua Dr. Roberto Frias, 4200-465 Porto, Portugal

**Keywords:** malnutrition, Mini-Nutritional Assessment (MNA), anthropometry, health conditions, Portugal, Turkey

## Abstract

Malnutrition is widespread among older adults, and its determinants may differ between countries. We compared Portuguese and Turkish non-institutionalized older adults regarding nutritional status, sociodemographic, health and anthropometric characteristics and studied the relationships between nutritional status and those characteristics. This cross-sectional study analyzed data from 430 Portuguese and 162 Turkish non-institutionalized older adults regarding sociodemographics, health conditions, the Mini-Nutritional Assessment (MNA-FF) and anthropometry. Turkish older adults were more likely to be malnourished or at risk of malnutrition and had lower average BMI but a higher calf circumference. A higher proportion of the Portuguese sample had tooth loss, diabetes, hypertension, oncologic diseases, kidney diseases, osteoarticular problems or eye problems, while less had anemia. A better nutritional status (higher MNA-FF score) was found among the Portuguese, males, people using dentures, those without tooth loss, hypertension, cardiovascular diseases, anemia or oncological diseases and was related to younger age, higher BMI and a higher calf circumference. Malnutrition and its risk were higher among older adults from Turkey, despite Portuguese older adults presenting a higher prevalence of chronic diseases. Being female, older age, tooth loss, hypertension, anemia, CVD or oncological disorders and having a lower BMI or CC were associated with higher rates of malnutrition among older adults from Portugal and Turkey.

## 1. Introduction

The older adult population (≥65 years) is increasing all over the world. Therefore, health measures for older adults are becoming more important, with poor nutritional status and malnutrition being major issues of concern. Malnutrition and unintentional weight loss contribute to a progressive decline in health, reduced physical and cognitive functional status, increased utilization of health care services and increased mortality. Despite that, clinicians and care givers are usually unaware of patients’ nutritional problems. Although malnutrition is widespread among geriatric patients, its diagnosis is usually underestimated; therefore, many do not receive appropriate treatment [1,2].

Malnutrition can be assessed using several tools. In this work, we used the Mini-Nutritional Assessment (MNA). The full form of the MNA (MNA-FF) was developed by Guigoz et al. (1994) to screen and assess malnutrition and its risk among older adults [3]. This instrument comprises 18 items that cover information regarding appetite or eating problems, recent weight loss, mobility impairment, acute illness/stress, dementia or depression, body mass index (BMI), living independently, the number of prescription drugs, pressure sores/skin ulcers, consumption of full meals, protein intake, fruit and vegetable intake, fluid intake, mode of feeding, self-perception of nutritional status, self-perception of own health status, mid-upper arm circumference (MUAC) and calf circumference (CC). The short form of the MNA (MNA-SF) considers only a subset of six questions from the MNA-FF, being frequently used as a screening tool [4,5].

The population of older adults differs between countries. These differences regard not only the proportion of older adults but also their characteristics, namely regarding malnutrition. Turkey and Portugal are an example, as they differ regarding socioeconomic and cultural features and present very different population characteristics of older adults. In Turkey, in 2017, older adults comprised 8.5% of the population [6], while they corresponded to 19.1% of the Portuguese population in 2011 [7]. With respect to nutritional status, a multicenter study in Turkey reported that, according to the MNA, 19.0% of community-dwelling older adults had malnutrition, and 29.1% were at risk of being malnourished [8]. In Portugal, also using the MNA, it was found that 1.3% of older adults were malnourished and 14.7% were at risk [9]. The cultural and socioeconomic differences between these two countries may lead to different determinants of the nutritional status. This has implications when tailoring public health interventions, namely when deciding which subgroups of older adults should be the focus of such interventions and when deciding on the applicability of the models and results of research carried out in different countries.

In order to better understand these differences and improve the quality of life of older adults, we conducted this study to compare Portuguese and Turkish non-institutionalized older adults regarding their nutritional status, sociodemographic, health and anthropometric characteristics and to study the relationships between nutritional status and sociodemographic, health and anthropometric characteristics.

## 2. Methods

The data used in this work were collected in 2015 (Turkey) and 2016 (Portugal) within two cross-sectional studies: the PRONUTRISENIOR project in Portugal and an older adult cross-sectional study in Turkey. The PRONUTRISENIOR project took place at the Family Health Unit (FHU) “Nova Via”, a primary care health center in Vila Nova de Gaia included in ACES Espinho-Gaia (Porto Metropolitan Area, Portugal) covering a heterogeneous population of older adults living in rural, semi-urban, urban, coastal and inland environments with different educational levels and socioeconomic status. Further details on this project are available elsewhere [10]. The Turkish sample was gathered from cross-sectional research conducted in both rural and urban areas of Ankara (capital city of Turkey), which included both community-dwelling and nursing home older adults, but only data from community-dwelling older adults were used in this study.

The inclusion criteria for both convenience samples were being at least 65 years old and being community-dwelling, i.e., non-institutionalized. The presence of dependency conditions (namely cognitive impairment) that could constrain free and informed decision-making regarding participation was used as an exclusion criterion.

Potential participants were contacted by phone or at the health care center. The acceptance to participate was given through signed informed consent. The study was approved by the Regional Administration of Health (approval number 2/2016) and was carried out in accordance with The Code of Ethics of the World Medical Association (Declaration of Helsinki).

Data were gathered using standard procedures by trained nutritionists and undergraduate nutrition students during their internship period via face-to-face interviews. Data collection took place at the primary health care centers attended by the participants or at their homes, according to their preference, and completed by phone.

In order to allow future comparisons with the results from different studies, both the MNA-SF and MNA-FF were used in the analysis. The MNA-SF includes six items: A—appetite or eating problems; B—recent weight loss; C—mobility impairment; D—acute illness/stress; E—dementia or depression and F—body mass index (BMI). The MNA-FF comprises these six items, plus: G—living independently; H—number of prescription drugs; I—pressure sores/skin ulcers; J—consumption of full meals; K—protein intake; L—fruit and vegetable intake; M—fluid intake; N—mode of feeding; O—self-perception of nutritional status; P—self-perception of own health status; Q—mid-upper arm circumference (MUAC) and R—calf circumference (CC) [4,5].

A better nutritional status corresponds to the absence of health problems (items A to E and I); higher anthropometric measurements (F, Q and R); living independently (G); lower number of prescription drugs (H); higher consumption or intake (J to M); oral feeding (N) and better perception of their own nutritional and health status (O and P). Items G, H, I, K, L, M, Q and R are scored 0 to 1; items A, C, D, E, J, N, O and P are scored 0 to 2 and items B and F are scored 0 to 3. The total scores correspond to the sum of the scores of the individual items (A to F for MNA-SF and A to R for the MNA-FF). For both forms, higher scores correspond to a better nutritional status. The MNA-FF score is categorized into malnourished (<17 points), risk of malnutrition (17 to 23.5) or normal nutritional status (24 to 30) [3]. The categories of the MNA-SF are similar: malnutrition (0 to 7 points), malnutrition risk (8 to 11) or normal nutritional status (12 to 14) [5].

In addition to completing the MNA-FF, all subjects in both surveys answered sociodemographic questions, which included sex, age, education level, household composition, marital status and professional situation. Health-related data were self-reported using a list of diseases and health conditions from which the participant should identify the ones with which they had been diagnosed. Anthropometric measurements were made following the standard methods [11,12,13,14,15] and included: weight, height, mid-upper arm circumference (MUAC), calf circumference (CC) and the computation of the body mass index (BMI).

The statistical analysis was performed using SPSS 22.0 for Windows. Descriptive statistics included the means and standard deviations for quantitative variables and relative (%) and absolute (n) frequencies for categorical variables. We applied Pearson’s chi-square tests to evaluate the independence between pairs of nominal variables and Student’s *t*-tests or Mann–Whitney’s *U* tests to compare, respectively, means and mean ranks between pairs of independent samples.

We performed an ANCOVA with the MNA-FF score as the dependent variable and the sociodemographic, anthropometric and health characteristics as independent variables. The initial model considered the following factors and covariables: country; sex; education level; marital status; professional situation; household composition; tooth loss; using dentures; having diabetes mellitus, hypertension, cardiovascular diseases, kidney diseases or osteoarticular diseases; age; BMI; MUAC and CC. Using a backward method, in each step, we removed the variable that had the least significant effect. We present the last step of this procedure, which includes only the significant relationships between each independent variable and the MNA-FF score, adjusted for the remaining variables included in that step. This analysis was sufficiently powered (80%) to detect an effect size (partial eta-squared; η_p_^2^) of 0.013 or larger.

Furthermore, we related each individual item of the MNA-FF with the sociodemographic, anthropometric and health variables using Spearman’s correlation coefficients and Mann–Whitney *U* tests. Variables with significantly higher (or lower) scores will be indicated with a (+) or (−) sign. In this analysis, we applied Bonferroni’s correction for multiple comparisons. All relationships were considered significant if *p* < 0.05.

## 3. Results

### 3.1. Sample Characterization and Comparison between Countries

In this study, we included a total of 592 older adults: 72.6% (*n* = 430) from Portuguese and 27.4% (*n* = 162) from Turkey. From the initial samples of 171 Turkish and 459 Portuguese participants, 9 cases were excluded from the Turkish sample and 29 from the Portuguese sample due to missing data. Table 1 presents and compares the Portuguese and Turkish samples regarding sociodemographic characteristics. We observed that Turkish older adults were more likely to have completed secondary school or to be illiterate, while Portuguese participants had mostly completed primary school. The Turkish sample had a higher proportion of participants living with children, who were a widow/widower and who were housewives/househusbands.

Portuguese older adults had a higher probability of having tooth loss, diabetes, hypertension, oncologic diseases, kidney diseases, osteoarticular problems or eye problems, while there was a higher frequency of anemia among Turkish participants. The mean BMI was smaller in the Turkish sample, while the mean calf circumference was larger when compared with the Portuguese sample (Table 2).

Regarding nutritional status, Turkish participants were more likely to be malnourished or at risk of malnutrition, both according to the MNA-SF and to the MNA-FF. However, the mean MNA score was significantly higher among Portuguese participants only for the MNA-SF (Table 3).

### 3.2. Relationships of Sociodemographic, Clinical and Anthropometric Characteristics with MNA-FF Scores

The final model of the ANCOVA is presented in Table 4. We observe that, on average, the MNA-FF score is higher among the Portuguese; males; participants using dentures and those without tooth loss, hypertension, cardiovascular diseases (CVD), anemia or oncological diseases and participants with younger ages, a higher BMI and higher CC. We note that the effect sizes (η_p_^2^) were larger for CVD and sex.

### 3.3. Relationships of Sociodemographic, Clinical and Anthropometric Characteristics with MNA-FF Items

Table 5 shows the relationships between each question that composes the MNA-FF and sociodemographic, health and anthropometric variables. Each reference category was chosen to match the one with the higher adjusted mean in the ANCOVA model. Most of the significant associations were in the same direction as those with the MNA-FF score in the ANCOVA model and are marked with a (+) sign. However, we also found some relationships in the opposite direction, marked with a (−) sign: older adults with higher BMI (question F) were more likely to have hypertension; those who took three or more prescription drugs (question H) were more likely to be Portuguese or to have higher BMI; those who took less full meals (question J) were more likely to be Portuguese, to have hypertension or to have higher BMI; Portuguese participants drank less fluids (question M) and regarding the perception of their health status (question P), Portuguese older adults felt worse.

## 4. Discussion

The main aims of this study were to compare Portuguese and Turkish non-institutionalized older adults regarding their nutritional status and to study its relationships with sociodemographic, health and anthropometric characteristics. We also compared the Portuguese and Turkish samples regarding their health and anthropometric characteristics, and, in order to better interpret those results, we analyzed the socioeconomic between-country differences. Moreover, we studied the relationships between those characteristics and each item of the MNA-FF.

### 4.1. Sample Characterization and Comparison between Countries

The prevalence of malnutrition and its risk was higher in the Turkish sample, while Portuguese older adults presented a higher proportion of tooth loss, diabetes, hypertension, oncologic diseases, kidney diseases, osteoarticular problems and eye problems. Additionally, Portuguese participants had higher BMI but lower CC, as well as a lower prevalence of anemia.

The sociodemographic differences found between Portuguese and Turkish older adults must be taken into account when defining public health interventions to prevent or treat malnutrition. Turkish older adults were more heterogeneous regarding their education level, which may imply some difficulties when defining communication strategies. On the other hand, the lower proportion of Turkish older adults living with their spouses seems to be somewhat equalized by living with their children or other relatives, as the proportion of participants living alone was similar in both countries.

In our study, Turkish community-dwelling older adults were more likely to be malnourished or at risk of malnutrition than the Portuguese ones (4.9% and 31.5% vs. 1.2% and 24.0%, respectively). The prevalence of malnutrition risk among European populations of older adults is about one-quarter, with lower proportions among community-living older adults [1,16,17] and, therefore, lower when compared to our results. On the other hand, a study among Portuguese older adults attending senior centers in Lisbon found a higher proportion (45.4%) of older adults malnourished or at risk of malnutrition when compared to both our Turkish and Portuguese samples, thus highlighting the relevance of considering specific sociodemographic characteristics [18].

Some results may be explained by inter-country economical and/or cultural differences. Portugal has a higher Gross Domestic Product (GDP) per capita than Turkey [19]. The GDP is positively correlated with the population’s BMI [20] and with the consumption of processed foods [21]. Tooth loss was more frequent among Portuguese participants, which may be due to a higher intake of sweets [22], which is also associated with a higher BMI [23]. The higher proportion of anemia among Turkish older adults may be related to different eating habits—in particular, a lower consumption of meat and meat products [24] and higher consumption of tea [25]—which may reduce iron absorption [26], together with their lower BMI [27].

In our study, Portuguese older adults had a higher BMI when compared to the Turkish. A higher BMI is related to a lower prevalence of malnutrition [28], but people with a high BMI are more likely to have chronic diseases, such as diabetes, hypertension, kidney diseases or CVD [29]. This is in line with the higher prevalence of such diseases in the Portuguese sample. Moreover, a complementary analysis showed that participants with BMI > 27 kg/m^2^ (n = 344, 58.1%) had a higher prevalence of diabetes (32.3% vs. 17.7%), hypertension (73.5 vs. 55.2%) and CVD (35.8% vs. 25.0%; *p* < 0.05 for all).

Portuguese older adults, when compared with the Turkish, presented lower CC, despite their higher BMI, which may be interpreted as lower muscular mass [30] and, consequently, higher fat mass. These results highlight the relevance of using different anthropometric measurements to assess older adults’ body compositions. Different patterns of physical activity may explain, at least partially, the opposite differences regarding BMI and CC, implying that more broad lifestyle approaches should be used to promote health among this age group.

Portuguese and Turkish participants differed regarding the MNA-SF but not the MNA-FF scores. This suggests that comparisons of the results using different instruments or different forms of the same instrument should be made cautiously. Older adults may be more or less at risk of malnutrition due to specific characteristics, namely dietary ones, as discussed below.

### 4.2. Relationships of Sociodemographic, Clinical and Anthropometric Characteristics with MNA-FF Scores

The MNA-FF score was higher among the Portuguese, males and younger older adults. Participants with dentures; without tooth loss, hypertension, CVD, anemia or oncological diseases and those with higher BMI and CC also presented a better nutritional status. Among these relationships, those with sex and CVD had the highest effect sizes.

Several studies in different regions are in line with female older adults being more likely to be malnourished [28,31,32,33]. Women have higher rates of depression and widowhood and a lower subjective health status, which are known risk factors for malnutrition [31]. Moreover, some reports show that women have significantly lower pension incomes than men [34,35]. In Europe, low income is associated with food insecurity, especially at low levels of social protection [36], and it is well known that food insecurity (i.e., limited or uncertain availability of nutritionally adequate and safe food) is a potential risk factor for malnutrition [37]. Therefore, sex-specific low income may be a cause of malnutrition as well. However, cultural differences on how much men and women are aware of dietary recommendations and on how dietary intake is related to chronic disease prevention or regarding cooking skills may also be relevant to interpret these results, as discussed by MacNab et al. (2018) when interpreting the lower nutritional risk among females in a sample of older adults in the USA [38].

CVD impose a substantial burden on the quality of life. Many patients with CVD have at least one other disease (such as diabetes or hypertension), which will have an even more negative effect on the quality of life [39,40]. Thus, CVD may cause a lower subjective health status and psychological stress or depression [41]. A study with Portuguese older adults attending senior centers showed reports that both CVD and lower self-reported health status were associated with higher nutritional risk [18], and the association between poorer perceived health status and higher risk of malnutrition has also been reported elsewhere [42]. In addition, for individuals with CVD, the MNA score may be affected due to the recommendation to reduce the consumption of red meat [43] if not replaced by other protein sources.

The finding that participants with dentures and without tooth loss presented higher MNA-FF scores is different from the one reported in a recent meta-analysis, which showed that edentulism was related to a higher risk of malnutrition among older adults and that the lack of teeth was a risk factor for malnutrition even when adjusting for sociodemographic variables [44]. However, the same meta-analysis reported no differences in the risk of malnutrition between dentate and edentulous people with two complete dentures. This may explain the difference from our results, as, in our sample, despite that we did not record the type (complete or not) of denture, from a total of nine out of ten older adults with tooth loss, about two-thirds had dentures.

### 4.3. Relationships of Sociodemographic, Clinical and Anthropometric Characteristics with MNA-FF Items

The relationships of the sociodemographic, clinical or anthropometric variables with some individual MNA items were in the opposite direction from those with the overall MNA-FF score. Taking more prescribed drugs (item H) was more common among participants with higher MNA-FF scores, and this may be related to a higher BMI and its associated comorbidities, which were more common among Portuguese older adults. Portuguese and Turkish participants seemed to differ regarding specific eating features, with the Portuguese consuming a lower number of full meals per day (item J, which does not imply that the overall food intake is smaller) and having a lower fluid intake (item M). These different characteristics may be one of the causes of a higher BMI and a higher likelihood of related comorbidities among the Portuguese sample, which may, in turn, result in a worse perception of one’s own health status. These assumptions are supported by the known relationships between these features (namely, the impact of obesity on health) but may also be due to cultural differences.

These results and previous knowledge [45,46] both support the conjecture that the Portuguese have less full meals, drink less fluids [47] and complain more about their health [48,49], namely when compared to Turkish older adults. However, the lack of studies directly analyzing the relationships of the sociodemographic, cultural, clinical and anthropometric characteristics with specific features related to malnutrition hardens the interpretation of such results, despite interest in the development of interventions.

### 4.4. Limitations and Strengths

Some limitations should be considered when interpreting the results of our study. The use of two convenience samples with different sociodemographic characteristics is one of them. However, the multivariate analysis allows the interpretation of the adjusted effects of the sociodemographic, clinical and anthropometric characteristics on the MNA-FF scores. The cross-sectional design of the study does not allow causality inference. The clinical data were self-reported and may therefore be biased, as there might be an underestimation of some diseases and health conditions due to a lack of memory.

On the other hand, to our best knowledge, this was the first study to compare and relate the nutritional status, health and demographic characteristics between Turkish and Portuguese older adults. We highlight the use of the same methodology and standardized procedures in the two samples. Additionally, the information about the relationships between specific malnutrition-related features (as assessed by the MNA items) and sociodemographic, clinical and anthropometric characteristics provides relevant information for the development of directed and precise interventions.

## 5. Conclusions

In conclusion, this study indicates that our sample of Turkish older adults living in the community was more likely to be malnourished than the Portuguese sample. However, Portuguese older adults presented a higher prevalence of chronic diseases. Moreover, being female, older age, tooth loss, hypertension, anemia, CVD or oncological disorders and having a lower BMI or CC were associated with higher rates of malnutrition among older adults from Portugal and Turkey.

## Figures and Tables

**Table 1:** Comparisons between countries regarding sociodemographic characteristics.

	Portuguese		Turkish		Total	
	Mean (SD)	Min; Max	Mean (SD)	Min; Max	Mean (SD)	Min; Max
**Age**, years (*p* = 0.362) ^t^	73.2 (5.8)	65; 94	72.7 (5.5)	65; 90	73.1 (5.7)	65; 94
	***n***	**%**	***n***	**%**	***n***	**%**
**Sex** (*p* = 0.850)						
Female	230	53.5	88	54.3	318	53.7
Male	200	46.5	74	45.7	274	46.3
**Education level** (*p* < 0.001)						
Illiterate	27	6.3	47	**29**	74	12.5
Did not complete primary school	47	10.9	36	22.2	83	14
Completed primary school	270	**62.8**	44	27.2	314	53
Attended secondary school	57	13.3	12	7.4	69	11.7
Completed secondary school or higher	29	6.7	23	**14.2**	52	8.8
**Who do you live with** (*p* = 0.013)						
Alone	72	16.7	29	17.9	101	17.1
With spouse	255	**59.3**	74	45.7	329	55.6
With spouse and children	55	12.8	30	**18.5**	85	14.4
With children or other relatives	48	11.2	29	**17.9**	77	13
**Marital status** (*p* = 0.003)						
Single	8	1.9	6	3.7	14	2.4
Married or similar	309	**71.9**	103	63.6	412	69.6
Divorced or similar	16	3.7	0	0	16	2.7
Widow/widower	97	22.6	53	**32.7**	150	25.3
**Professional situation** (*p* < 0.001)						
Active	18	4.2	5	3.1	23	3.9
Retired or unemployed	400	**93**	89	54.9	489	82.6
Housewife/househusband	12	2.8	68	**42**	80	13.5

Pearson’s chi-square test, except for ^t^ Student’s *t*-test.

**Table 2:** Comparisons between Portuguese and Turkish older adults regarding health and anthropometric characteristics.

	Portuguese	Turkish	Total
	*n (%)*	*n (%)*	*n (%)*
**Tooth loss** (*p* < 0.001)	**399 (92.8)**	129 (79.6)	528 (89.2)
**Denture** (*p* = 0.867)	266 (61.9)	99 (61.1)	365 (61.7)
**Diabetes Mellitus** (*p* < 0.001)	**144 (33.5)**	11 (6.8)	155 (26.2)
**Hypertension** (*p* < 0.001)	**319 (74.2)**	71 (43.8)	390 (65.9)
**Cardiovascular diseases** (*p* = 0.086)	143 (33.3)	42 (25.9)	185 (31.3)
**Anemia** (*p* = 0.005)	24 (5.6)	**20 (12.3)**	44 (7.4)
**Oncological diseases** (*p* = 0.016)	**34 (7.9)**	4 (2.5)	38 (6.4)
**Kidney diseases** (*p* = 0.006)	**51 (11.9)**	7 (4.3)	58 (9.8)
**Osteoarticular diseases/problems** (*p* < 0.001)	**236 (54.9)**	55 (34.0)	291 (49.2)
**Eye diseases/problems** (*p* = 0.001)	**407 (94.7)**	20 (12.3)	427 (72.1)
	**Mean (SD)** **[Min; Max]**	**Mean (SD)** **[Min; Max]**	**Mean (SD)** **[Min; Max]**
**Body mass index**, kg/m^2^ (*p* < 0.001) ^U^	**29.2** (4.7) [18.4; 51.8]	26.6 (4.9) [17.6; 54.1]	28.5 (4.9) [17.6; 54.1]
**Mid Upper Arm Circumference**, cm (*p* = 0.534) ^U^	29.3 (3.6) [19.0; 55.0]	29.6 (5.5) [15.0; 57.0]	29.4 (4.2) [15.0; 57.0]
**Calf Circumference**, cm (*p* < 0.001) ^t^	35.8 (3.5) [21.5; 47.5]	**38.2** (6.3) [23.0; 60.0]	36.4 (4.6) [21.5; 60.0]

Pearson’s chi-square tests, except ^t^ Student’s *t*-tests or ^U^ Mann–Whitney *U* test.

**Table 3:** Comparisons between Portuguese and Turkish older adults regarding nutritional status.

	Portuguese		Turkish		Total	
	*n*	%	*n*	%	*n*	%
**MNA-SF classification** (*p* < 0.001)						
Malnourished (0 to 7 points)	3	0.7	11	**6.8**	14	2.4
Risk of malnutrition (8 to 11 points)	45	10.5	57	**35.2**	102	17.2
Normal nutritional status (12 to 14 points)	382	88.8	94	58	476	80.4
**MNA-FF classification** (*p* = 0.003)						
Malnourished (<17 points)	5	1.2	8	**4.9**	13	2.2
Risk of malnutrition (17 to 23.5 points)	103	24	51	**31.5**	154	26
Normal nutritional status (24 to 30 points)	322	74.9	103	63.6	425	71.8
	**Portuguese**		**Turkish**		**Total**	
	**Mean (SD)**	**Min; Max**	**Mean (SD)**	**Min; Max**	**Mean (SD)**	**Min; Max**
**MNA-SF score** (*p* < 0.001) ^U^	13.1 (1.4)	6.0; 14.0	**11.5** (2.1)	4.0; 14.0	12.7 (1.8)	4.0; 14.0
**MNA-FF score** (*p* = 0.437) ^U^	24.6 (2.0)	15.5; 28.5	24.0 (3.7)	11.5; 30.0	24.4 (2.6)	11.5; 30.0

Pearson’s chi-square tests, except ^U^ Mann–Whitney *U* test. MNA-SF: Mini-Nutritional Assessment—Short Form; MNA-FF: Mini-Nutritional Assessment—Full Form.

**Table 4:** Relationships between MNA-FF scores and sociodemographic, clinical and anthropometric variables (ANCOVA).

	Mean	Adjusted Mean	SD	η_p_^2^	*p*
Overall				0.211	<0.001
**Country**				0.027	<0.001
Portugal	24.6	**23.9**	2		
Turkey	24	22.9	3.7		
**Sex**				0.045	<0.001
Females	24	22.9	2.8		
Males	25	**24**	2.2		
**Tooth loss**				0.012	0.008
No	25.1	**23.9**	2.5		
Yes	24.4	23	2.6		
**Denture**				0.007	0.049
No	24.5	23.2	2.5		
Yes	24.4	**23.7**	2.6		
**Hypertension**				0.022	<0.001
No	24.8	**23.8**	2.8		
Yes	24.2	23.1	2.4		
**Cardiovascular diseases**					
No	24.8	**24**	2.3	0.056	<0.001
Yes	23.5	22.8	2.9		
**Anemia**					
No	24.6	**24**	2.4	0.018	0.001
Yes	22.8	22.8	3.6		
**Oncological diseases**					
No	24.5	**24**	2.6	0.015	0.003
Yes	23.5	22.9	2.7		
	**R**		**η_p_^2^**		***p***
**Age**	**−0.111**		0.011		0.012
**Body mass index**	**+0.049**		0.01		0.017
**Calf circumference**	**+0.115**		0.012		0.007

MNA-FF: Mini-Nutritional Assessment—Full Form; R: Pearson’s correlation coefficients; η_p_^2^: partial eta-squared.

**Table 5:** Relationship of each MNA full form item with the sociodemographic, health and anthropometric variables.

	A	B	C	D	E	F	G	H	I	J	K	L	M	N	O	P	Q	R
**Portugal**		+	+	+	+	+		−		−	+	+	−	+	+	−	+	
**Males**	+			+														
**Age (low)**	No significant relationship found with the individual items.																	
**BMI (high)**						+		−		−								+
**CC (high)**						+												+
**No tooth loss**	No significant relationship found with the individual items.																	
**Yes denture**	No significant relationship found with the individual items.																	
**No hypertension**						−		+		−						+		
**No CVD**								+								+		
**No anemia**					+				+									
**No oncological diseases**	No significant relationship found with the individual items.																	

MNA full form items: (A) appetite or eating problems; (B) recent weight loss; (C) mobility impairment; (D) acute illness/stress; (E) dementia or depression; (F) body mass index (BMI); (G) living independently; (H) number of prescription drugs; (I) pressure sores/skin ulcers; (J) consumption of full meals; (K) protein intake; (L) fruit and vegetable intake; (M) fluid intake; (N) mode of feeding; (O) self-view of nutritional status; (P) self-perception of own health status; (Q) mid-upper arm circumference (MUAC) and (R) calf circumference (CC). The (+) or (−) sign indicates that the variables on the left have significantly higher (or lower) scores for the corresponding MNA item. Spearman’s correlation coefficients: items A, B, C, E, F, J, K, M, N, O, P and Q. Mann–Whitney’s *U* test: items D, G, H, I, L and R. Only significant relations after Bonferroni’s correction are marked. Each reference category was chosen to match the one with the higher adjusted mean in the ANCOVA.

## Data Availability

The datasets used in the current study are available from the corresponding author upon reasonable request.
